# Association of multiple chronic conditions and pain among older black and white adults with diabetes mellitus

**DOI:** 10.1186/s12877-017-0652-8

**Published:** 2017-10-30

**Authors:** Tamara A. Baker, Olivio J. Clay, Vicki Johnson-Lawrence, Jacquelyn A. Minahan, Chivon A. Mingo, Roland J. Thorpe, Fernando Ovalle, Michael Crowe

**Affiliations:** 1 Department of Psychology, University of Kansas, Lawrence, KS USA; 2 Department of Psychology, University of Alabama at Birmingham, Birmingham, AL USA; 3 Department of Public Health and Health Sciences, University of Michigan-Flint, Flint, MI USA; 4 Gerontology Institute, Georgia State University, Atlanta, GA USA; 5 Bloomberg School of Public Health, Johns Hopkins University, Baltimore, MD USA; 6 Division of Endocrinology, Diabetes and Metabolism, University of Alabama at Birmingham, Birmingham, AL USA

**Keywords:** Multiple chronic conditions, Pain, Older adults, Diabetes, Determinants of health

## Abstract

**Background:**

Aging is often associated with the challenge of navigating daily tasks with a painful chronic medical illness. Yet, there is concern of the number of older adults impacted with more than one chronic condition. Despite the increasing number of adults diagnosed with diabetes and comorbid chronic illnesses, there remains a lack of understanding in how multiple illnesses relate to experiences of pain. To assess the association between multiple chronic conditions and pain, this study aimed to identify clusters of chronic medical conditions and their association with pain among a sample of older Black and White adults diagnosed with diabetes.

**Methods:**

Two hundred and thirty-six participants responded to a series of questions assessing pain frequency and severity, as well as health and social characteristics. A factor analysis was used to categorize clusters of medical conditions, and multiple regression models were used to examine predictors of pain.

**Results:**

Seven of the assessed chronic medical conditions loaded on three factors, and accounted for 57.2% of the total variance, with heart disease (factor 1) accounting for 21.9%, musculoskeletal conditions (factor 2) for another 18.4%, and factor 3 (microvascular diseases) accounting for a final 16.9% of the variability among the chronic medical conditions. Covariate-adjusted models showed that fewer years of education and higher scores on the microvascular and musculoskeletal conditions factors were associated with higher pain frequency, with the musculoskeletal conditions factor being the strongest predictor.

**Conclusions:**

Findings from this study compliment existent literature underscoring the prevalence and importance of comorbid diagnoses in relation to pain. Examining health-related factors beyond a single disease diagnosis also provides an opportunity to explore underlying disease co-occurrences that may persist beyond organ system classifications.

## Background

The Centers for Disease Control (CDC) estimates that more than half (54%, 117 million people) of all persons in the US have at least one chronic disease, with three-in-four adults 65 years of age and older reporting two or more conditions [1]. Non-communicable diseases, also known as chronic diseases, tend to be long in duration and are the result of environmental, genetic, psychological, and behavior factors.^2^ With this definition, there is the emergent association of increasing age and being diagnosed with multiple chronic conditions (MCC) [2–4]. Importance of this construct is underscored by the increase in the number of adults diagnosed with more than five chronic illnesses from 22% to 35% in 2006 and 2010 [5].

The Agency for Healthcare Research and Quality data show that 71% of total health care expenditures is associated with care for those with MCC.^5^ Similarly, those diagnosed with a chronic medical illness also report increased use of health care services (over 133 million dollars) compared to those with an acute illness. These multiple diagnoses account for 76% of all physician visits, 81% of hospital admissions, and 91% of all prescriptions filled. Yet, with these expended costs, discussion remains not only of the onset and management of chronic illnesses, but the consequences these conditions have on the daily lived experiences of those diagnosed with a chronic disease [6].

Epidemiological evidence suggests that cardiovascular diseases (e.g., myocardial infarctions, stroke), cancers, chronic respiratory diseases (e.g., chronic obstructed pulmonary disease, asthma), diabetes, obesity, and arthritis are the most common chronic conditions, and often preventable medical conditions [7]. These illnesses are related to disability and mortality, with heart disease and cancer diagnoses collectively accounting for nearly 48% of all reported deaths in the US [8].

### Pain and MCC dyad

Despite findings underscoring the relevancy and financial strain caused by comorbid diagnoses, there remains concern about the increasing number of individuals diagnosed with multiple painful chronic illnesses. While positioned as a serious health concern, pain has traditionally been defined as a physiological response to disease and tissue damage, and is often classified into different categories based on its origin: nociceptive (tissue damage or inflammation), neuropathic (nerve damage), and mixed or unspecified (unknown causes or a combination of nociceptive and neuropathic) [9]. Yet, these categorizations do not encompass the social, environmental, and cultural factors that influence the pain experience. More importantly, little attention has focused on the (multiple) chronic illness-pain dyad, particularly among the aged population. This is all the more challenging when addressing the needs and health outcomes among those diagnosed with a specific medical illness, such as diabetes.

Recent data show that an estimated 10.9 million of older American adults are diagnosed with diabetes, with an expected increase to 26.7 million by the year 2050 [10]. US Data sources show that in 2014, the age-adjusted prevalence of diabetes among Black adults, 18+ years of age, was 13.4 compared to 7.3 for Whites [11]. The confirms findings from the US Department of Health and Human Services, finding that Black adults are 80% more likely than Whites to be diagnosed with diabetes, and 4.2 times more likely to be diagnosed with end stage renal disease compared to White adults [12].

Aside from these statics, diabetes is associated with several comorbidities (e.g., diabetic retinopathy, sleep apnea, neuropathy, nephropathy), with an overwhelming majority of research studies suggesting robust associations between diabetes and cardiovascular disease, and diabetes and pain (i.e., heart disease and stroke) [13].

Pain is a major healthcare problem and is often considered an underlying symptom of a disease process. Although accepted as a symptom of a disease diagnosis or injury, the recurrence of pain can also be considered a disease in itself. Existent data suggest that while many patients are diagnosed with a primary pathology (e.g., arthritis, diabetes), the changes to the peripheral nervous system may have demonstrable secondary pathology. Thus, the individual may then develop persistent pain as a disease, whereby it meets the criteria for a disease entity having its own pathology and symptoms [14, 15]. This secondary pathology may have implications in understanding the association of pain and diabetes. Pain onset among those diagnosed with diabetes may be more severe and sudden, with pain also being experienced in the chest, stomach, side, and the outside of the shin or inside of the foot [16]. Particularly when dealing with older diabetic patients with MCC, it is important to recognize that pain associated with the chest and abdomen are often mistaken as somaticized pain for heart disease or a heart attack [16]. This issue is critical as adults with diabetes are four times more likely to have heart disease or a stroke compared to those who do not have the diagnosis [3]. The intersection of diabetes and other comorbid conditions would presumably heighten the medical complexity for older adults, which makes caring for the them particularly arduous.

While the pain and a single chronic condition (e.g., cancer, diabetes, hypertension), relationship has received overwhelming attention among the general population, evidence defining the clustering of MCC and pain dyad among older adults is less evident. To contribute to our understanding of pain-related MCC among older adults, this study aimed to categorize (cluster) diagnosed chronic conditions, and to assess the association of categories of MCC with pain (frequency, severity) among a sample of older Black and White patients with diabetes. Determining the influence of multiple comorbid conditions, beyond that of a single medical condition and the pain experience, is a strength of this study.

## Method

### Participants

Data were collected as part of the baseline assessment for the University of Alabama at Birmingham (UAB) Diabetes and Aging Study of Health (DASH). The aim of the UAB DASH project was to examine potential disparities between older African American and White older adults with diabetes [17, 18]. Participants included community-dwelling older adults from Birmingham, Alabama and surrounding areas, as well as patients from a diabetes clinic at UAB. All participants were 65 years of age and older and had either self-identified or was physician diagnosed with diabetes. Respondents were not required to self-report pain to be enrolled in the study.

Community-dwelling participants were recruited from a commercially available list (maintained by the UAB Roybal Center for Translational Research on Aging and Mobility) of older adults in the Birmingham metropolitan area. Clinic participants were recruited from patients of a co-investigator at the UAB Diabetes and Endocrinology Clinic. All participants were contacted via mail followed by telephone contact. The study oversampled Black adults due to the overarching goal of the study to examine racial disparities in mental health, cognitive function, and mobility outcomes in older adults with diabetes.

## Measures

### Pain severity and frequency

Pain was measured with single-item questions assessing severity and frequency. Participants were asked, “How frequently over the past four weeks have you experienced pain?” Response choices included: not at all (0), less than once a week (1), 1–3 times a week (2), 4–6 times a week (3), and daily (4). Respondents were similarly asked to rate their pain severity on a five-point Likert scale: no pain (0), annoying (1), uncomfortable (2), dreadful (3), and agonizing (4). Investigators added the EQ-5D quality of life scale which also has a single-item pain/discomfort severity assessment at follow-up. The pain severity item described above had a strong correlation with the frequently used item from the EQ-5D, *r* = 0.62, *p* < .0001. This provides evidence of the utility of the pain severity item.

### Health variables

#### Chronic health conditions

Chronic medical diagnoses were assessed using a list of commonly occurring chronic health conditions. Participants self-reported if they had been told by a doctor or nurse that they had any of the following chronic conditions: osteoporosis, renal or kidney disease, stroke, hypertension, heart attack or myocardial infarction, congestive heart failure, and arthritis. Response choices were coded as either no (0) or yes (1).

### Demographic characteristics

A total of six demographic variables were assessed for purposes of this investigation. Age was assessed in years, and education was assessed as the highest grade completed (grades 1–12, 14 = Associates Degree or some college, 16 = Bachelor’s Degree, 18 = Master’s Degree, 20 = M.D. or PhD). Race was self-reported as White or Black. Income was measured in ordinal categories ranging from 1 (less than $5000) to 9 ($100,000 or greater). Marital status assessed percent married.

### Statistical analyses

All statistical analyses were conducted using SAS V9.1.3. Descriptive statistics were calculated to provide details on measures and a profile of the sample’s demographic (age, sex, race, education, income, marital status), health (comorbidities), and pain (frequency and severity) characteristics. African American and White adults were compared using the chi-square independence test for categorical measures and independent samples t-tests for continuous measures to examine group differences on variables of interest.

An exploratory factor analysis was performed to identify clusters of medical conditions and to reduce the number of variables included in subsequent multiple regression models. Criteria of an examination of the scree plot and eigenvalues greater than one were used to select the number of factors to retain. A varimax rotation (orthogonal) was chosen rather than oblique rotation due to the investigators’ goal to produce factors of medical conditions that were as distinct from each other and not correlated [19]. Standardized factor scores, with a mean of 0 and a standard deviation of 1, were extracted for the clusters of conditions retained from the results of the factor analysis. Linear multiple regression models were utilized to examine covariate-adjusted associations between variables of interest including the factor scores for the clusters of medical conditions and pain frequency and severity in separate models. The race x sex interaction term was also assessed in these models.

## Results

### Demographic and health characteristics

The sample consisted of 236 older adults, with 47% (*n* = 110) self-identifying as Black. For the total sample, the Black participants were predominately female, younger, reported less years of formal education, earned less income, less likely to be married, and were more likely to self-report being diagnosed with hypertension (*p-values < .02*). No other bivariate racial differences emerged between the racial groups. Other demographic characteristics are reported in Table 1. Participants recruited from the UAB Diabetes and Endocrinology Clinic were more likely to report renal or kidney disease, heart attack or myocardial infarction, and congestive heart failure than those recruited from the community (*p*’s < .05).

**Table 1:** Descriptive statistics for study variables

Measures	Total Sample (*n* = 236)	African American (*n* = 110)	Caucasian (*n* = 126)	*p*-value
Age, mean (std)	73.40 (6.11)	72.39 (5.67)	74.28 (6.38)	0.0177
Education, mean (std)	13.44 (2.63)	12.95 (2.68)	13.87 (2.53)	0.0078
Income, mean (std)	5.01 (1.92)	4.22 (1.69)	5.70 (1.84)	<0.0001
Married, *n* (%)	112 (47.46)	38 (34.55)	74 (58.73)	.0002
Female sex, *n* (%)	125 (52.97)	69 (62.73)	56 (44.44)	.0050
Osteoporosis, *n* (%)	37 (15.68)	12 (10.91)	25 (19.84)	.0597
Renal or kidney disease, *n* (%)	29 (12.29)	13 (11.82)	16 (12.70)	.8372
Stroke, n (%)	13 (5.51)	6 (5.45)	7 (5.56)	.9729
Hypertension, *n* (%)	193 (81.78)	98 (89.09)	95 (75.40)	.0066
Heart attack or myocardial infarction, *n* (%)	38 (16.10)	14 (12.73)	24 (19.05)	.1876
Congestive heart failure, *n* (%)	35 (14.83)	14 (12.73)	21 (16.67)	.3956
Arthritis, *n* (%)	170 (72.03)	78 (70.91)	92 (73.02)	.7191
Pain frequency, mean (std)	2.21 (1.59)	2.22 (1.55)	2.20 (1.63)	.9244
Pain severity, mean (std)	1.53 (1.14)	1.60 (1.15)	1.48 (1.14)	.4074

The seven assessed chronic medical conditions loaded on three latent factors, accounting for 57.2% of the total variance of the medical conditions assessed. As reported in Table 2, Factor 1 was labeled “Heart Disease”, included congestive heart failure, heart attack (myocardial infarction), and stroke, and this factor accounted for 21.9% of the total variability of chronic medical conditions. Factor 2 was labeled “Musculoskeletal Conditions” (osteoporosis and arthritis) and accounted for 18.4% of the variability among the chronic medical conditions. The third factor, “Microvascular Diseases,” (renal or kidney disease and hypertension) accounted for another 16.9% of the variability.

**Table 2:** Factor loadings for 3 Factor solution with varimax rotation

Measures	FACTOR 1: Heart disease and cancer	FACTOR 2: Musculoskeletal conditions	FACTOR 3: Microvascular diseases
Congestive heart failure	*0.7841*	0.0229	0.1264
Heart attack or myocardial infarction	*0.7119*	0.0086	0.0615
Stroke	*0.5832*	0.0227	−0.1197
Osteoporosis	−0.0514	*0.7984*	−0.1349
Arthritis	0.1239	*0.7278*	0.1927
Renal or kidney disease	0.1741	−0.1876	*0.7745*
Hypertension	−0.1557	0.2892	*0.7014*
Eigenvalue	1.534	1.287	1.181
Percent of Variance Accounted for	21.9	18.4	16.9

Italicized values refer to a medication condition loading on a particular factor

As shown in Table 3, with the covariate-adjusted models, fewer years of education (B = −0.22), higher scores on the microvascular diseases factor (B = 0.15), and higher scores on the musculoskeletal conditions factor (B = 0.31) were associated with higher pain frequency (*p-values < .05*).

**Table 3:** Covariate-adjusted associations between variables of interest and pain frequency

Variables	B	b	t-statistic	*p*-value
Age	−0.084	−0.022	−1.33	.1843
Education	−0.222	−0.134	−3.05	.0026
Income	0.049	0.041	0.56	.5772
Married	−0.036	−0.116	−0.51	.6134
Female sex	−0.095	−0.301	−1.01	.3140
African American Race	−0.198	−0.629	−2.09	.0380
African American Race x Female sex Interaction	0.276	0.962	2.43	.0160
Heart Disease	0.088	0.140	1.44	.1503
Musculoskeletal Conditions	0.313	0.497	4.70	<.0001
Microvascular Diseases	0.147	0.235	2.40	.0174

**Fig. 1:**
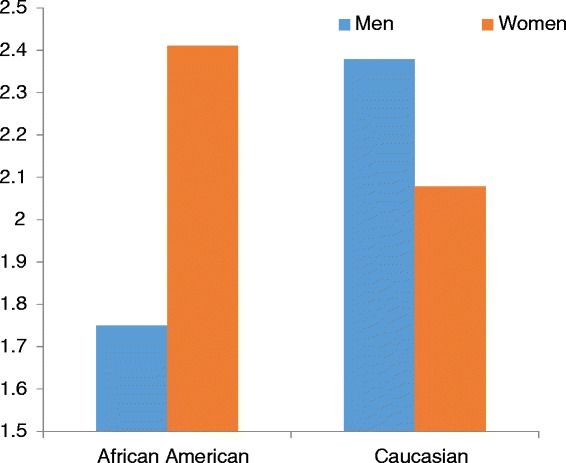
Race x Sex interaction on Pain Frequency

Additionally, a race x sex interaction showed that Black men reported less pain frequency than the other groups (B = 0.28, *p* = .02; Fig. 1). When pain severity was assessed, fewer years of education (B = −0.22) and higher scores on the heart disease (B = 0.19), musculoskeletal conditions (B = 0.19), and microvascular diseases (B = 0.17) factors were each individually associated with higher levels of pain severity, p’s < .01 (Table 4).

**Table 4:** Covariate-adjusted associations between variables of interest and pain severity

Variables	B	b	t-statistic	*p*-value
Age	−0.113	−0.021	−1.80	.0731
Education	−0.218	−0.095	−3.02	.0028
Income	−0.063	−0.038	−0.73	.4679
Married	0.030	0.068	0.42	.6753
Female sex	0.070	0.160	0.75	.4528
African American Race	−0.046	−0.106	−0.49	.6234
African American Race x Female sex Interaction	0.036	0.091	0.32	.7486
Heart Disease	0.187	0.214	3.09	.0023
Musculoskeletal Conditions	0.187	0.214	2.83	.0050
Microvascular Diseases	0.169	0.193	2.77	.0061

Notes: B = Standardized beta, b = unstandardized beta

## Discussion

While there is much emphasis on “healthy living and healthy aging”, there is growing public health concern regarding the deleterious impact chronic, and often painful and debilitating, medical diagnoses have on the well-being and quality of life of an aging adult. While empirical data have focused on singular medical conditions, the sciences have generally failed to recognize the impact of MCC diagnoses among the aging adult population. This is not to fault prior empirical findings, but rather acknowledge the need to produce findings that guide the need to further understand the physical, behavioral, social, and economic impact of MCC.

Results from this study are interesting in that there were three distinct groupings of chronic conditions among this sample of older adults with diabetes. While there were significant peripheral differences between the two race groups in reports of chronic conditions and other demographic characteristics, what should be noted is the effect size of the musculoskeletal conditions factor in relation to pain. While not surprising, this finding directs our attention to the significant number of older persons (inpatient and outpatient) dealing with the social, mental, and physical consequences of a musculoskeletal condition. This is especially relevant as 47% and 51% among those 65–74 and 75 and over, respectively, are diagnosed with an arthritis-related illness.^8^ With arthritis being recognized as the nation’s number one cause of disability [20], there is the need to better understand the short- and long-term implications of this diagnosis, particularly among those diagnosed with arthritis and another chronic medical illness (e.g., diabetes, hypertension, etc.). For example, 49% of adults diagnosed with arthritis also have heart disease, with another 47% being diagnosed with comorbid diabetes [20]. Yet, these diagnoses do not speak to the myriad of issues that are experienced among those diagnosed with a painful chronic medical illness. Considered a common problem for many patients with arthritis, and more recently considered a medical diagnosis of its own, chronic pain is particularly salient to understanding the complications of being diagnosed and living with MCC.

Similarly, finding that participants recruited from the UAB Diabetes and Endocrinology Clinic were more likely to report renal or kidney disease, heart attack or myocardial infarction, and congestive heart failure than those recruited from the community, was expected considering that these individuals were probably more sick, and/or experiencing more complicated health outcomes, thus requiring them to see a diabetes specialist. This is consistent with current data showing that clinic-based adults tend to be more ill than community-based adults.

An amalgam of data illustrate the robust association for the chronic illness and pain association [21–26]. Existent data establishes the impact pain episodes have on the capacity to maintain an independent lifestyle [25, 26]. This process characterizes the multidimensionality of the pain-chronic illness association, thereby augmenting how chronic disease is defined, particularly among the aged population. In addressing the association between pain and chronic illnesses, results from this study also provide interesting findings in that Black males reported less pain frequency than the other groups. Reports show that men are often socialized to project strength, individuality, autonomy, and avoid expression of emotion or vulnerability; all of which could be interpreted as weakness [27–29]. These socially contrived characteristics may have influenced the Black male participants’ responses to the pain questions, thereby not wanting to appear vulnerable [29]. This coincides with the historical context of the Black males’ existence in the US, where showing signs of weakness could have devastating social, mental, and physical health implications. This explanation contends with existing evidence suggesting that Black men who report pain have a higher risk for greater pain severity and affective disorders [30]. This finding clearly shows the need for more research to better understand the pain experience among this population.

Conventional focus on single diagnoses and outcomes may negatively affect symptom management and treatment adherence, and has substantial implications for healthcare models. While chronic health conditions are all too common in older adults, they also have serious social, physical, and behavioral implications for the well-being of individuals across a continuum of demographic characteristics. Understanding the biologic and social influences of these factors are important to consider if the institutional health care paradigm is to adequately diagnosis, screen, and treat diverse and marginalized patient populations. For example, results from this study (and many others) show that Black participants were less likely to be formally educated and more likely to be diagnosed with hypertension. These results show that race is a commonly recognized indicator that has economic and health implications. Race is a complex and multidimensional social construct that establishes a conceptual framework for how we perceive health, illness, social demands, and environmental changes across the life course.

Cultural factors also have a pronounced influence on health by affecting exposure and vulnerability to disease, risk-taking behaviors, clinical presentation, course and outcome of the illness, and access to and availability of quality health care. To understand the role of race in one’s lived experiences, is to understand the extent to which the individual identifies with that particular race (or cultural) group, and how events and situations throughout the life course influence these identified behaviors.

The Joint Center for Political and Economic Studies [31] suggests that by eliminating health disparities in chronic disease management, there would be a reduced direct medical cost by $229.4 billion, and a reduced indirect-related cost, associated with the illness and premature mortality, by approximately $1 trillion. This underscores the compendium of existent data describing the reality of racial disparities in health status and chronic disease diagnoses. These disparities are often perpetuated by social indicators such as income, education, and neighborhood and built environment, and have a significant impact not only among those from diverse race populations, but also across multiple social identities, all of which may influence the onset and outcome of MCC diagnoses.

### Influence of social determinants of health on MCC outcomes

To account for this epidemic of health disparities, there is a growing interest to identify and understand the underlying reasons for these disparities. Findings from this investigation support the need to better understand why there may be these defined race differences, and how social and behavioral scientist should direct their attention to further understanding the underlying causes of MCC diagnosed differences. Termed by the CDC and WHO, social determinants of health (SDoH) provide an explanation for not only the onset of a chronic illness, but more importantly, the circumstances by which these illnesses exist. Advocated by Healthy People 2020, these determinants define systems, whereby people are born, grow up, live, work, and age [32, 33]. The WHO further cites social determinants as being multifaceted, integrated, and overlapping social structures and economic systems that identify upstream and downstream factors that impact chronic disease diagnoses and management.^32^ The details of SDoH can amass over a lifetime causing fluctuations in health trajectories across the life course. For example, neighborhood environments with access to healthy foods, quality housing, reduced exposure to crime and violence, and better environmental conditions (i.e., green space) show improved health outcomes. SDoH addresses indicators beyond the individual as a patient with the chronic illness, but rather provides meaning of the circumstances of the individual before, during, and after the diagnosis. The influence of determinants of health concurs with recent empirical data showing the influence of race and economic status on the chronic pain experience, with patients with fewer years of education, and Black adults residing in lower social economic neighborhoods, reporting increased pain disability and mood disorders [34].

The role of SDoH can never be overstated. Understanding their influence may bring about a better knowledge of health outcomes, and how best to indigenize treatments for chronic illnesses across multiple populations and the life course. An understanding of these determinants of health implies that we can achieve better health outcomes by addressing the circumstances of the individual by promoting (prevention) intervention-based practices and self-management strategies, whereby the individual can achieve optimal symptom management, while improving quality of life. Improvement may result from early detection and diagnosis, medication adherence, adequate health care access and resources, advanced technology, and universal healthcare (insurance).

Although this study showed interesting findings in the clustering of certain chronic medical illness and the association with the pain experience in those with diabetes, there are a few study limitations that must be acknowledged. First, the study did not include a standardized measure of pain. Though pain presence and severity were assessed, the two single-itemed questions may not have fully captured the pain experience of the patient population. Similarly, due to the cross-sectional design of the study, it was difficult to determine the cause of pain. Although all participants were diagnosed with diabetes, along with other chronic illness, there was no accurate measure to determine the exact cause of pain, whether it was from the diabetes diagnosis, musculoskeletal condition, or some other pain-related medical condition.

Another limitation was that the results were based on data exclusively from older adults from one southern state. This limits the generalizability to other regions, minority groups, and those younger than 65 years of age. Also, because chronic illnesses were self-reported, there was no method to confirm the health diagnosis. This may lead to disease misclassification, thereby influencing the clustering analyses. The data were collected via self-reports and may result in potential reporting bias (e.g., social desirability). However, prior studies have found that reliability of self-reported diabetes is very high compared to information from general practitioners as well as medical records [35, 36]. The overall sample size for the current investigation and the sample size to the number of variables ratio were adequate to assess the factor structure for the conditions assessed [37]. Nevertheless, additional studies with larger sample sizes are needed to produce more stable estimates.

## Conclusions

Findings from this study provide a number of implications for clinical research. Recognizing the increasing number of older adults diagnosed with more than one chronic illness is important when addressing overall treatment and disease management. Focus should be on treating the patient as a whole rather than ‘piecing’ together treatment plans, by various medical specialists. Lack of communication among patients and multiple specialists may result in an overlap in treatment and decreased medical adherence. This may be particularly important for patients with diminished cognitive capacities. In terms of better understanding the determinants of health, more measurement work is needed in interpreting pathways by which different social factors contribute to the overall (positive/negative) health outcomes of the adult population. This may provide a better understanding of how chronic illnesses (and related symptoms) are diagnosed, treated, and managed.

In assessing the needs of older adults diagnosed with multiple medical illnesses, it is necessary to provide individuals with the skills needed to effectively manage their comorbid diagnoses. Considerable efforts have been made to address issues surrounding the management of MCC, particularly among underserved and marginalized individuals. It is important that public health initiatives focus on reducing the burden of chronic disease for all and minimizing health disparities, while redirecting society’s priorities to the benefits of evidenced-based health prevention and management programs. Chronic disease self-management programs (CDSMP), for example, introduce skills that may allow chronically ill individuals the ability to self-manage their medical conditions. There is also growing evidence of the benefits of these programs in reaching vulnerable older adults [38], rural populations [39], and specific medical conditions (e.g., arthritis) [40, 41]. Yet, what must be clearly understood is that MCC diagnoses are embedded in health-related outcomes that reflect a biological, behavioral, and social patterning of differential treatment, rights, and privileges that are defined by the life-course, and are surrounded by larger historical, geographic, social, cultural, and economic milieus. These constructs outline important factors such as socioeconomic positioning, contextual grounding of race, sex, and marginalized populations, and explanations for similarities and differences in health outcomes. This may help to identify factors that have important implications for public policy, advocacy, and long-term medical needs of a diverse aging population.

## Abbreviations

MCC: Multiple Chronic Conditions
SDoH: Social Determinants of Health
CDC: Centers for Disease control
WHO: World Health Organization
CDSMP: Chronic Disease Self-Management Programs
DHHS: US Department of Health and Human Services
NIDDK: National Institute of Diabetes and Digestive Kidney Diseases.
